# The role of cognitive stimulation at home in low-income preschoolers’ nutrition, physical activity and body mass index

**DOI:** 10.1186/s12887-017-0918-5

**Published:** 2017-08-01

**Authors:** Saskia Op den Bosch, Helena Duch

**Affiliations:** 1 SEO Scholars, New York, NY USA; 2 Mailman School of Public Health, Columbia University, New York, NY USA; 3 Mailman School of Public Health, Department of Population and Family Health, Columbia University, 60 Haven Avenue, B-2, New York, NY 10032 USA

## Abstract

**Background:**

Early childhood obesity disproportionately affects children of low socioeconomic status. Children attending Head Start are reported to have an obesity rate of 17.9%.This longitudinal study aimed to understand the relationship between cognitive stimulation at home and intake of junk food, physical activity and body size, for a nationally representative sample of 3- and 4-year old children entering Head Start.

**Methods:**

We used The Family and Child Experiences Survey 2006. Cognitive stimulation at home was measured for 1905 children at preschool entry using items from the Home Observation Measurement of the Environment Short Form. Junk food consumption and physical activity were obtained from parent interviews at kindergarten entry. BMI z scores were based on CDC national standards. We analyzed the association between early cognitive stimulation and junk food consumption, physical activity and BMI, using multinomial and binary logistic regression on a weighted sample.

**Results:**

Children who received moderate levels of cognitive stimulation at home had a 1.5 increase in the likelihood of consuming low amounts of junk food compared to children from low cognitive stimulation environments. Children who received moderate and high levels of cognitive stimulation were two and three times, respectively, more likely to be physically active than those in low cognitive stimulation homes. No direct relationship was identified between cognitive stimulation and BMI.

**Conclusion:**

Prevention and treatment efforts to address early childhood obesity may consider strategies that support parents in providing cognitively stimulating home environments. Existing evidence-based programs can guide intervention in pediatric primary care.

## Background

Childhood obesity has more than tripled in the last 30 years, with a prevalence of 8.4% among children ages 2 to 5 [1]. In addition, obesity disproportionately affects children of low socioeconomic status, with a rate of nearly 15% for children under the age of 5 [1] in this group. Children who are obese have a greater chance of being obese during adulthood, increasing the likelihood of serious health conditions such as heart disease, stroke, type 2 diabetes and various forms of cancer [2]. It is well established that obesity is a result of complex interactions between genetic, environmental, and social factors [3, 4]. One current model proposes six levels of contributors: cellular, child, clan, community, country and culture [5]. For young children, the clan or family level may be of particular importance as young children spend most of their time at home [6].

Within the clan/family level, one intriguing factor—parental stimulation of the child’s cognitive development (e.g. opportunities for play and learning)—has been linked to the prevention of overweight and obesity. Strauss & Knight [7], using a nationally representative sample of U.S. children, identified a greater than two-fold increase in the risk of developing obesity for children exposed to low levels of cognitive stimulation in their early home environment. Additionally, work of Garasky and colleagues, [8] investigating a variety of family stressors and their influence on body mass index (BMI) outcomes in children, supported a positive association between lack of cognitive stimulation and child overweight and obesity. While these studies point to cognitive stimulation in the early home environment as an important influence on the development of obesity, the mechanisms by which the home environment may be associated to body size in childhood are still largely unknown.

In the preschool years, parents have significant control over their children’s nutrition [9–13] and opportunities for physical activity, [14–17] both of which significantly influence obesity. Therefore, this study examines the association between cognitive stimulation at home, nutrition, physical activity and Body Mass Index (BMI z score). While previous work has documented a relationship between cognitive stimulation in the home and body mass index, our work extends this prior literature but examining the relationship between cognitive stimulation and more proximal outcomes, like junk food consumption and physical activity.

Given that obesity in early childhood is almost double among low income children, [1] our study focuses on participants in the federally funded Head Start program, which reports an obesity rate of 17.9% and an overweight rate of 19.9% among participating children, [18] making this a crucial population to study in understanding contributors to early childhood obesity in America.

We hypothesize, first, that independent of socio-demographic factors, moderate to high levels of cognitive stimulation in the home at preschool entry will be associated with higher levels of physical activity and lower levels of junk food consumption at the end of kindergarten. Second, based on the results of Strauss and Knight [7], we hypothesize that lower levels of cognitive stimulation in the home at preschool entry will be associated with higher body mass index (BMI) at the end of kindergarten. The findings from this study can be used to inform the development of interventions that consider the impact of home influences on young children’s nutrition and physical activity practices, and ultimately on body size.

## Methods

### Participants

The Family and Child Experiences Survey dataset (FACES, 2006) was used for this study [19]. There are five FACES cohorts (1997, 2000, 2003, 2006 and 2009), each including a nationally representative sample of 3- to 4- year-old children entering Head Start for the first time. Data are collected on children and families at three time points in the span of 2 years: 1) fall of child’s first year in Head Start, 2) spring of the same year, and 3) spring of the following year. This study focuses on the baseline (2006) and the last two waves of data collection (2008 and 2009). Sample size was 1905 children and their families (see Table 1 for demographics) and includes almost equal numbers of enrolling 3- (51%) and 4-year olds (49%). Most of the sample was Hispanic (37%), with comparable numbers of Whites (24%) and African-Americans (30%). Thirty nine percent of mothers had less than a high school diploma. Seventy-two percent of participants reported a household income below 30,000 dollars per year. A majority of the sampled children (85%) had normal birth weight. Analysis of de-identified data was used for this study and it was exempt from Columbia University’s Institutional Review Board. A restricted license for FACES 2006 was obtained for work on this manuscript.

**Table 1:** Demographic characteristics of sampled children and their families^ab^

Demographics	Total number (of Sampled children)	Percent of sample
Gender		
Male Female	991 914	52.0% 48.0%
Race/ Ethnicity		
White Black Hispanic Other	457 572 705 171	24.0% 30.0% 37.0% 9%
Maternal Education		
Less than High School High School or GED^c^ More than High School	743 591 572	39.0% 31.0% 30.0%
Household Income		
Less than 30 K Greater than 30 K	1372 533	72.0% 28.0%
Marital Status		
Not Married Married	1238 667	65.0% 35.0%
Birth Weight^d^		
Normal Weight (>5.5lbs)	1619	85.0%
Cohort		
% 3-year olds % 4-year olds		51.0% 49.0%

^a^ *N* = 1905 weighted sample

^b^Updated at Follow-up, 2009

^c^General Education Diploma

^d^Excludes cases of birthweight >5.5 lbs

### Predictor variable

#### Cognitive stimulation

To develop a cognitive stimulation composite variable from the questions used in the FACES 2006 parent interview, we matched the FACES 2006 items [19] to questions used in the Home Observation Measurement of the Environment-Short Form (HOME-SF), a nationally-recognized, standardized measure for assessing the home environment of young children [7]. After matching, a total of 22 items were selected and re-coded to match the coding for the HOME-SF and summed to create a composite variable (see Table 2). As per the HOME-SF scoring and following the work of Strauss & Knight, [7] cutoff points were created at the 15th and 85th percentiles and categorized as: 0–11 = 1 (low cognitive stimulation), 12–17 = 2 (medium cognitive stimulation), and 18–23 = 3 (high cognitive stimulation).

**Table 2:** Coding key for cognitive stimulation composite questions^a^

Question	Coding
Frequency read to child in past week	0 = less than 3× / 1 = 3× or more
No. of minutes/day child is read to	0 = less than 20 min / 0 = more than 20 min
Told them a story	0 = no / 1 = yes
Taught child letters, words, numbers	0 = no / 1 = yes
Taught child songs or music	0 = no / 1 = yes
Worked on arts and crafts with child	0 = no / 1 = yes
Took child on errands	0 = no / 1 = yes
Involved child in household chores	0 = no / 1 = yes
Talked about what happened in Head Start	0 = no / 1 = yes
Talked about TV programs/videos	0 = no / 1 = yes
Played counting games	0 = no / 1 = yes
Visited a library with child	0 = no / 1 = yes
Gone to a movie with child	0 = no / 1 = yes
Gone to a play or concert with child	0 = no / 1 = yes
Gone to a mall with child	0 = no / 1 = yes
Visited art gallery or museum	0 = no / 1 = yes
Visited zoo or aquarium with child	0 = no / 1 = yes
Talked with child about heritage	0 = no / 1 = yes
Attend community sponsored event	0 = no / 1 = yes
Attended church activity/school	0 = no / 1 = yes
Number of children books in household	0 = less than 10 / 1 = more than 10

^a^Composite variable created as a sum of these items

### Outcome variables

#### Junk food consumption

Food consumption was evaluated using parental reports of consumption of junk food per week, obtained during the third and fourth follow-up interviews (in 2008 and 2009, respectively). Four categories of junk food were defined in creating a junk food score: sugary snacks; cookies, cakes and brownies; fast food; and salty snacks. Answers were scored on a scale of 0 to 5 depending on frequency of consumption. The score on the 0–5 rating scale was then re-coded into categories (no consumption, low consumption, moderate consumption, high consumption) based on cut-offs at the 15th and 85th percentile, where low consumption meant that a child did not have junk food more than 3 times in 1 week, and moderate consumption meant the child did not have junk food more than once a day. Information on consumption of healthy foods was not available in this dataset.

#### Physical activity

To create a dichotomous variable of physical activity level, information on whether a parent took the child to participate in a game/sport/exercise in the past week (1 = yes, 0 = no) and whether parent took child to a playground/park (1 = yes, 0 = no) was added and coded as 1 = active and 0 = not active, where active meant that parent had engaged the child in both activities in the past week.

#### BMI Z scores and categories

We based our BMI z scores and categories on the Center for Disease Control (CDC) national standards for children ages 2 to 5, based on height, weight, gender and age in months [20]. BMI z scores were generated using the STATA commands *zanthro()* and *zbmicat()* which take as their primary argument a child’s BMI composite, available in the FACES 2006 dataset [19]. Scores for BMI are categorized into 1 = normal weight, 2 = overweight and 3 = obese, where z scores above the 85th percentile are categorized as overweight and above the 95th percentile as obese, using a BMI-for-age reference chart in the US [20].

### Analysis

All analyses were weighted using the FACES 2006 weight, PRA16WT, and conducted in STATA 10 using the statistical package svyset() and the Taylor-Series method to adjust variances. To test bivariate relationships between study variables, cross tabulations and chi-square tests of independence were performed on the weighted sample (*n* = 1905).

The association between early cognitive stimulation and consumption of junk food (dependent variable) at follow up was analyzed using multinomial logistic regression, converting log odds to relative risk ratios for each level of the dependent variable, and controlling for socio-demographic variables.

The association between early cognitive stimulation and physical activity levels at follow up (dependent variable) was estimated using binary logistic regression analyses, adjusting for socio-demographic factors (maternal education, child birth weight, race, age, and gender).

Because the majority of the sample (Table 1) was in the same category of household income (72% below $30,000) and marital status (65% not married), these variables were dropped from the analyses, as they did not improve the explanatory power and significance of the overall model.

## Results

Table 3 summarizes the distribution for the outcome and predictor variables of interest. The majority of children (65%) had medium levels of cognitive stimulation at home while approximately 18% had low or high levels. The majority of children were not active (71%) and consumed moderate amounts of junk food (58%). The percentage of overweight children was between 16 and 21% throughout the study years, while the percentage of obese children ranged between 6 and 14%.

**Table 3:** Descriptives for predictor and outcome variables of interest^ab^

Descriptive	Fall 2006	Spring 2008	Spring 2009
Cognitive Stimulation			
% Low Cognitive	17		
% Medium Cognitive	65		
% High Cognitive	18		
Physical Activity			
% Active		29	28
% Not Activ		71	72
Junk Food Consumption			
% Low Consumption		38	39
% Moderate Consumption		58	56
% High Consumption		3	4
BMI Category			
% Normal Weight	76	70	71
% Overweight	18	16	21
% Obese	6	14	8

^a^ *N* = 1905 weighted sample

^b^0.01% and 0.02% of sample reported no consumption of junk food in 2008 and 2009, respectively

Our hypothesis that lower levels of cognitive stimulation in the home at preschool entry is associated with BMI z score at the end of kindergarten was not supported by the data: cross tabulation between cognitive stimulation and BMI categories did not yield a significant statistic after a chi-squared test of independence of variable distribution (*p* > 0.05). Multiple logistic regression confirmed this lack of significance with effect sizes for cognitive stimulation that were not significant [F(12,36) = 12.33, −.007, *p* = 0.462] before and after controlling for demographic factors.

Our hypotheses that moderate to high levels of cognitive stimulation at preschool entry are associated with lower levels of junk food consumption and with higher levels of physical activity at the end of kindergarten were partially supported by the data (Table 4). Specifically, for the relationship between cognitive stimulation and junk food consumption, a multinomial logistic regression, adjusted for socio-demographic factors, showed that children who received *moderate cognitive stimulation* at baseline (fall 2006) had a 1.5 increase (*p* < 0.05) in the likelihood of consuming low amounts of junk food in the spring of 2008, compared to children residing in environments with low cognitive stimulation. Results indicated that high levels of cognitive stimulation at home were not associated with consuming low amounts of junk food (*t* = 1.28, *p* = 0.207).

**Table 4:** Relative risk of low junk food consumption predicted by cognitive stimulation in 2008 [F(39,9) = 319.7, *p* = 0.000]

	Relative risk ratio and 95% CI of low junk food consumption
Medium Cognitive Stimulation^a^	1.5^*^ (1.02, 2.29)
Maternal Education > High School^b^	1.5^**^ (1.08, 2.20)
*Additional covariates in model:*	
*Birth weight*	0.96 (0.88, 1.07)
*Race:* ^*c*^	
*African American*	0.70 (0.47, 1.03)
*Hispanic/Latino*	1.0 (0.73, 1.37)
*American Indian/ Alaska native*	0.60 (0.21, 1.64)
*Asian or Pacific Islander*	2.57 (0.51, 13)
*Multiracial*	1.3 (0.63, 2.77)
*Other race*	0.70 (0.21, 2.39)
*Age*	0.99 (0.97, 1.02)
*Gender*	0.80 (0.61, 1.03)

^*^ *p* < 0.05, ^**^ *p* < 0.02

^a^compared to low cognitive stimulation

^b^compared to less than high school

^c^compared to White/Non Hispanic

Analysis could not be performed for the 2009 follow up because data for the nutrition composite variable was not available at that time point. In addition, maternal education above high school level associated inversely with junk food consumption at the 2008 follow up (*p* < 0.01).

Regarding the relationship between cognitive stimulation and physical activity, binary logistic regression revealed that children who were categorized as having *moderate* cognitive stimulation at baseline were twice (*p* < 0.0001) as likely to be physically active at the 2008 follow-up as those with *low* cognitive stimulation at home (Table 5). When children had *high* cognitive stimulation at baseline, the odds of being active increased further, i.e. to three times (*p* < 0.0001) that of those with low cognitive stimulation. This effect for children with high cognitive stimulation at baseline remained in the 2009 follow up, when the chance of being physically active was two and a half times (*p* < 0.005) that of children with low cognitive stimulation at baseline. Together these data indicate that higher levels of cognitive stimulation are positively associated with physical activity, and that the effect may persist over at least the short term.

**Table 5:** Odds of being physically active predicted by cognitive stimulation

	2008 Odds ratio of being active OR [95%CI] (F = 5.11)^****^	2009 Odds ratio of being active OR [95%CI] (F = 2.74)^**^
Medium Cognitive Stimulation^a^	2.0^****^ (1.46,2.81)	1.42 (0.89, 2.26)
High Cognitive Stimulation^a^	2.8^****^ (1.87,4.27)	2.67^**^ (1.36,5.25)
*Additional covariates in model:*		
*Maternal education*		
*High School/ GED*	1.04 (0.77, 1.40)	1.33 (0.83, 2.12)
*More than High School*	0.74 (0.53, 1.03)	0.94 (0.54, 1.65)
*Race*		
*African American*	1.62^**^ (1.13, 2.32)	1.47 (0.73, 2.95)
*Hispanic/Latino*	2.00^**^ (1.25,3.32)	1.61 (0.84, 3.09)
*American Indian/ Alaska native*	0.43^***^ (0.25, 0.74)	0.31 (0.93, 1.02)
*Asian or Pacific Islander* ^*b*^	3.89^*^ (1.08, 14.0)	
*Multiracial*	2.70^*^ (1.17, 6.38)	2.05 (0.48, 8.76)
*Other race*	1.30 (0.31, 5.52)	1.75 (0.24, 12.83)
*Age*	1.00 (0.98, 1.02)	1.06 (0.99, 1.12)
*Gender*	1.09 (.087, 1.36)	1.24 (0.87, 1.76)

^*^ *p* < 0.05, ^**^ *p* < 0.01, ^***^ *p* < 0.001, ^****^ *p* < 0.0001

^a^compared to low cognitive stimulation

^b^Data for Asian Pacific Islander was omitted in 2009

In the 2008 follow up, race ethnicity was positively associated with physical activity for African American, Hispanic, Asian and Multi Racial children (compared to Whites/non Hispanic) and negatively associated to physical activity for American Indian/Alaska Native children. These relationships were not observed in the 2009 follow up.

## Discussion

This study explored the relationship between the home cognitive environment, nutrition, physical activity and body size in a national sample of preschool aged children attending Head Start. Analysis of the cognitive stimulation composite variable showed that moderate levels of cognitive stimulation at home at preschool entry were associated with lower levels of junk food consumption. In addition, moderate and high levels of cognitive stimulation at home were associated with higher levels physical activity in kindergarten. We also tested for an association of cognitive stimulation with BMI, but unlike previous studies, [7, 8] we did not find a direct association between cognitive stimulation, as measured by items in the HOME scale short-form, [7] and young children’s BMI. Differences in our sample (low-income children) and some items in the HOME-SF may account for these differences.

The results of our study inform the design of early childhood obesity prevention and intervention efforts by highlighting an important target area, cognitive stimulation at home, and its possible implications for improved physical activity and nutrition outcomes.

### Physical activity and cognitive stimulation

We identified a positive relationship between cognitive stimulation and physical activity.

We measured cognitive stimulation with items from the well-established and widely used HOME-SF scale [21]. While some of the items in the HOME scale do relate directly to promoting physical activity in children (e.g. visiting zoos, running errands), the vast majority of items do not support this construct and involve what typically are sedentary behaviors (e.g. reading books, teaching letters and numbers). Therefore, the relationship between parental responses to the HOME scale and increased physical activity requires further exploration.

Studies that have examined the influence of the home environment on preschoolers’ physical activity have identified the availability of toys that promote activity (as well as backyard equipment), parents’ own physical activity, parental monitoring of television use, the presence of other children and, verbal prompts to be physically active as positive influences on physical activity [14–17]. A next step to understanding the relationship between cognitive stimulation and physical activity promoting behaviors would be to systematically study home influences and resources for physical activity and to characterize these factors in assessment measures of the home environment.

### Junk food consumption and cognitive stimulation

Our study identified an inverse relationship between moderate levels of cognitive stimulation and junk food consumption. The relationship between these constructs is likely mediated by parental behaviors that are associated with children’s eating habits [9, 11–13]. In support of this, prior research has identified maternal food intake, parenting practices and attitudes to be associated with young children’s diet [9, 11–13]. Next steps to understanding the relationship between cognitive stimulation at home and junk food consumption should explore how the suggested mediating effect of parental factors operates.

Consistent with prior research, we found an association between maternal education and child feeding behavior. Parents with less than a high school education were more likely to have children who consumed higher levels of junk food. Hendricks and colleagues [10] found that having a college degree was associated with breastfeeding and positive child feeding behavior, advocating for targeted interventions for parents with lower levels of education.

### Opportunities for intervention

Many current efforts to reduce obesity in early childhood have focused on improving children’s diets, encouraging physical activity and improving community practices that support healthy lifestyles (access to food, parks, safety…). Our results suggest another potential type of intervention—improving cognitive stimulation in the home by providing resources and activities that parents can engage in with their children to support their overall development.

Indeed, a significant number of evidence-based programs nationally and internationally are focusing on improving parent–child interactions and supporting early childhood development. Some of these interventions are delivered through home visiting such as the Nurse Family Partnership [22] or Early Head Start, [23] and others through center-based interventions or public health and media campaigns such as “Too Small to Fail [24]”.

In addition, pediatricians and the primary care practice have been home to the promotion of positive cognitive stimulation in the home. Successful and promising programs like “Reach out and Read,” [25] “Healthy Steps,” [26] and the “Video Interaction Project (VIP)” [27] amongst others, have been adept at promoting positive parent–child interactions and cognitive stimulation in the home. Such evidence-based programs provide information, skill building and consultation for families, arming them with a range of tools to encourage their young children’s development. Based on the results of our study, promoting cognitive stimulation at home is also critical to impact healthy nutrition and physical activity practices, particularly for low income children and children whose mothers have less than a high school education. Building on evidence-based models, pediatricians may engage families through referrals to home and center based programs, or through practice-based interventions that may range in intensity from a reading campaign such as “Reach out and Read” [25] to more comprehensive models that use developmental specialists integrated in primary care.

### Limitations and strengths

While prior work that examined the relationship between cognitive stimulation and children’s BMI used the HOME short form scale, [7] to achieve our proposes we had to create a cognitive stimulation composite by matching available items in the FACES parent interview to those in the HOME. Although items in both sets did not differ significantly (See Table 2), our measure may not have been as sensitive at capturing the home cognitive environment as the HOME-SF [21]. This possible resulting lack of sensitivity may have contributed to the difference between our results and those of Strauss and Knight [7]. However, differences in measurement may not fully account for the lack of findings since we also studied a different population. In addition, FACES 2006 [19] did not contain information on healthy eating practices and we therefore had to focus our nutrition variable on junk food consumption. Finally, information on physical activity was limited to parental reports based on a few broad questions on children’s engagement in park/ recreational activities and participation in games, sports and exercise.

Despite these limitations, our study has significant strengths. In exploring the relationship between nutrition, physical activity and cognitive stimulation, we expanded our understanding of the relationship between early cognitive stimulation, junk food consumption and physical activity. We used a sample of low income children with a high incidence of overweight and obesity, which further informs the design of interventions that target obesity promoting behaviors and practices with high-risk populations.

## Conclusion

Efforts to address and prevent early childhood obesity need to consider interventions to help parents and caregivers provide learning and cognitively stimulating home environments for children. The absence of these opportunities may negatively influence children’s physical activity and nutritional intake, and ultimately their body size. A wide range of evidence based programs, delivered through home visiting, center based and pediatric practices have successfully targeted the home environment to improve child development outcomes. These programs should be extended to address young children’s obesity risk. Future research should examine specific parenting and child characteristics that may influence the relationship between the home cognitive environment, nutrition and physical activity.

## Abbreviations

BMI: Body Mass Index
FACES: The Family and Child Experiences Survey
HOME-SF: The Home Observation for Measurement of the Environment Short Form
VIP: The Video Interaction Project
